# 

**Published:** 1902-04-12

**Authors:** 


**Notices of Births, Deaths, and Marriages** are inserted in this column at a charge of 2s. 6d. for an announcement not exceeding four lines.

**DEATH.**

March 27th, after three weeks' illness, Nurse Sara Johns, General Infirmary, Gloucester. Much regretted. (410)

### NOTICE TO CORRESPONDENTS.

All MSS., Letters, Books for Review, and other matters intended for the Editor should be addressed The Editor, "The Hospital," The Hospital Building, 28 & 29 Southampton Street, Strand, W.C.

The Editor cannot undertake to return rejected MS., even when accompanied by stamped directed envelope.

**Notice to Advertisers and Subscribers.**

All Advertisements, Orders for Copies of The Hospital, and Business Communications should be addressed to **THE MANAGER, (Not to the Editor)**, The Hospital, 28 & 29 Southampton Street, Strand, London, W.C.

The Cost of Subscription, post free, is as follows.—Per week, 2½d.; per quarter, 2s. 8d.; per half-year, 5s. 3d.; per annum, 10s. 6d. Foreign:—Per annum, 15s. 2d., payable, in all cases, in advance.

**NOTICE.—Vol. XXXI. of "The Hospital" (October 1901, to March 1902), in handsome cloth binding will shortly be on sale at the office of this Journal, price, 7s. 6d. Cases for binding the Numbers contained in this Volume 1s. 6d. Index to Vol. XXXI., 2½d., post free.**

**The Monthly Part for April is now ready. Price 6d., by post 8d.**

### BOOKS RECEIVED.

Chatto and Windus.

"Herbert Fry's Royal Guide to the London Charities." Edited by John Lane.

S. Harvey, Earlstown.

"Annual Report of the Medical Officer, Haydock Urban District, for 1901." By T. E. Hayward, M.B.Lond.

Scientific Press.

"Burdett's Hospitals and Charities, 1902. Being the Year-Book of Philanthropy and the Hospital Annual." By Sir Henry Burdett, K.C.B.

Archibald Constable and Co.

"From Cradle to School: a Book for Mothers." By Mrs. Ada S. Ballin.

John Wright and Co.

"The Medical Annual: a Year-Book of Treatment and Practitioners' Index, 1902." Twentieth Year.

### Scraps and Gleanings.

The London Shoe Company, Limited, have been appointed by royal warrant shoemakers to H.R.H. the Princess of Wales.

A convalescent home for officials connected with the Belfast and Northern Counties Railway Company was recently opened at Whitehead. The building contains dining, drawing, and smoke-rooms, and beds for six visitors. Considerable expense has been incurred by Mrs. S. P. Kerr (who originated the idea of starting this institution), but further funds are required if the home is to be a success.

The number of patients treated in the Cumberland Infirmary during the past year amounted to 955 in- and 2,642 out-patients, and is the largest number yet treated at this infirmary in any one year. The expenditure exceeded the income by £1,300. A bazaar is to be arranged for next year, and by this means it is hoped that there will be realised a sum sufficient to place this institution on a sounder financial footing.

### The Student of Medicine.

### Appointments.

**A. M. Barford**, M.D., L.R.C.P., M.R.C.S., appointed Assistant Anæsthetist to the Royal Dental Hospital, Leicester Square. **Jane Boyes**, L.R.C.P., L.R.C.S.Edin., L.F.P.S.Glasg., appointed Medical Officer for the Island of Coll, Argyllshire. **N. C. Boyle**, L.R.C.P., L.R.C.S Edin., appointed Medical Officer of Health by the Penge Urban District Council. **C. E. Corlette**, M.D.Syd., appointed Acting Honorary Assistant Surgeon to the Women's Hospital. Sydney, during the absence of Sir James Graham. **T. G. Davy**, M.R.C.S., L.S.A.Lond., appointed Honorary Medical Officer to the Fremantle Hospital, West Australia. **E. G. Leger Erson**, L.&L.Mid.R.C.F.Edin., appointed Officer of Health, Peak Hill, West Australia. **Arthur Evans**, M.S.Lond., F.R.C.S.Eng., appointed Assistant Surgeon to Westminster Hospital. **A. Greenwood**, M.D.Vict., appointed Medical Officer of Health of Blackburn. **James A. T. Hall**, L.R.C.P., L.R.C.S.Edin., L.F.P.S.Glasg., appointed Medical Officer and Public Vaccinator of the Stoke Golding District of the Hinckley Union. **Mitchell Hunter**, M.D. Miami Med. Col., L.S.A.Lond., appointed Certifying Surgeon under the Factory Act for the Magherafelt District of the County of Londonderry. **W. M. Hunter**, M.B., B.S., R.U.I., appointed Certifying Surgeon under the Factory Act for the Crumlin District of Antrim. **Harold Lister**, M.B.Melb., appointed Acting Medical Superintendent of the Ararat Lunatic Asylum, Victoria, during the absence on leave of W. H. Barker, M.R.C.S.Eng. **F. S. Lloyd**, M B.Lond., appointed Certifying Surgeon under the Factory Act for the Luton District of Bedfordshire. **James Thomas Mitchell**, M.D., Ch.M.Aberd., M.R.C.S., appointed Resident Medical Officer to the Ballarat Benevolent Asylum and Lying-in Home, Victoria. **J. C. McLean**, M.B., C.M.Edin., appointed Medical Officer of Health by the Doncaster Rural District Council. **T. E. Pallett**, L.S.A.Lond., appointed Certifying Surgeon under the Factory Act for the Earl's Colne District of Essex. **James Pearse**, M.D., C.M.Edin., appointed Medical Officer of Health of Trowbridge, Wilts.

**THE MENOPAUSE AND ITS DISORDERS.**

With Chapters on Menstruation.

BY

A. D. LEITH NAPIER, M.D., M.R.C.P.,

Late Editor of the *British Gynæcological Journal.*

Royal 8vo., cloth gilt, Illustrated by a series of Original Photo-micrographs and many Illustrations in the Text, **7/6** net.

"Of great practical use to the general practitioner as well as to those more specially engaged in gynæcological work."—*The Hospital.*

THE SCIENTIFIC PRESS, LTD.,
28 & 29 Southampton Street, Strand, W.C.

